# Improving Understanding of Reflexivity in Family Medicine: Development of an Educational Tool Based on a Rapid Review

**DOI:** 10.15694/mep.2021.000181.1

**Published:** 2021-07-19

**Authors:** Marie-Claude Tremblay, Laurence Garceau, Ndeye Thiab Diouf, Anne Guichard, Julien Quinty, Chantal Gravel, Christian Rheault

**Affiliations:** 1 Université Laval

**Keywords:** Reflexivity, Reflective practice, Medical Education, Health professional training, Rapid review

## Abstract

This article was migrated. The article was marked as recommended.

**Background:** In the last decade, reflexivity has emerged as a key concept in family medicine, as evidenced by its increasing integration in competency statements and frameworks in the field. However, the concept of reflexivity is inconsistent and ill-defined in medical education literature, with variable purposes and associated processes, which is an important barrier to learning and implementing reflective practices. This project built on the results of a rapid review to develop an educational tool supporting the learning and teaching of reflexivity in family medicine.

**Methods:** We conducted a rapid review of quantitative, qualitative and mixed studies relating to reflexivity in family medicine between May 2007 to May 2017 in *PubMed, Embase, PsychInfo, CINHAL, ERIC* and *Education Source.* Two reviewers independently identified, selected and reviewed studies. Results of the review were used to frame the content of the tool.

**Results:** Our research strategy initially identified 810 studies, from which 65 studies were retained for analysis. The different conceptions of reflexivity encountered in the included studies were analyzed using thematic analysis. Four conceptions of reflexivity (i.e. clinical, professional, relational and social reflexivity), with related definitions, goals and processes were identified in the included studies and were used as a basis to develop the Reflexivi-Tool.

**Conclusion:** There is a need to provide clear guidelines regarding the purpose and process of reflexivity, as well as better equipping mentors so they can better facilitate these kinds of skills. Based on a rapid review, this study has allowed the development of a tool that presents and clarifies four main types of reflexivity for medical practice in a concise and user-friendly way. Tools such as Reflexivi-Tool are crucial to support reflective processes that target different dimensions of professionalism.

## 1. Background

Reflexivity is a concept emerging from social sciences that has generated a lot of interest in a wide range of disciplines, such as education, sociology, organizational sciences, psychology, nursing and medicine ( D’Cruz, Gillingham and Melendez, 2007; Mann, Gordon and MacLeod, 2009). Generally conceived as an alternative form of professional learning rooted in experience and reflection on experience, reflexivity appears to be a crucial component of responsible professional practice and its continuous improvement ( Mezirow, 1990; Issitt, 2003; Gustafsson and Fagerberg, 2004; Mann, Gordon and MacLeod, 2009).

In medicine, reflexivity is conceived as a cross-cutting competence that enables health care providers to build new knowledge repertoires from their experiences, to deal with complex and unusual situations in their practice, and to adapt to changes in the health system by promoting continuous learning and adaptation ( Mann, Gordon and MacLeod, 2009; Sandars, 2009; Wald and Reis, 2010; Menard and Ratnapalan, 2013). Reflexivity, reflection and reflective practice are concepts promoted in a wide variety of competency statements at all levels of medical education, both nationally and internationally. For instance, the Accreditation Council for Graduate Medical Education competencies (US), the CanMEDS-MF competency framework (Canada) and the European Federation of Internal Medicine evoke reflective practice in relationship with the development of clinicians’ core competencies, such as professionalism ( Tannenbaum, 2009; Frank, 2015). According to the CanMEDS-FM, reflexive clinicians demonstrate “awareness of self and an understanding of how one’s attitudes, beliefs, assumptions, values, preferences, feelings, privilege and perspective impact their practice”, and reflect “on practice events, especially critical incidents, to deepen self-knowledge and recognize when something needs to change and does it” (p.18). The ability to think critically and learn from experience is also described by Mann, Gordon and MacLeod (2009) as a key element that differentiates the novice from the expert in medical practice and the exercise of his clinical reasoning.

An important challenge to understanding reflexivity is that terminology and definitions of this concept are highly variable in literature, hindering the concept’s full application in training and professional practice ( Mann, Gordon and MacLeod, 2009; Chaffey, de Leeuw and Finnigan, 2012). For instance, our review of reflexivity in medical education (described in this article) found “reflection”, “self-directed learning”, “habits of mind”, “reflective practice”, “reflective learning”, “mentalization” and “insightful practice” as variable terms related to reflexivity with more or less consistency ( Bethune and Brown, 2007; Laidlaw *et al.*, 2007; Davidsen and Reventlow, 2011; George *et al.*, 2013; Al-Imari, Yang and Pimlott, 2016). A commonly cited definition of reflexivity is that of Sandars, which sees reflection (i.e. reflexivity) as “a metacognitive process that occurs before, during and after situations with the purpose of developing greater understanding of both the self and the situation so that future encounters with the situation are informed from previous encounters” ( Sandars, 2009, p. 685). As for Chaffey, de Leeuw and Finnigan (2012), they view reflection in medicine as “the action of thinking critically and consciously about one’s practice, so as to reduce the risk of non-conscious habitual practice, which can lead to compromised patient care and safety” (p. 198). If processing of experience to inform future action appears as a recurrent feature of many definitions of reflexivity, the purposes and goals of this type of activity vary greatly: reflexivity is sometimes considered as an approach to develop a therapeutic relationship or professional expertise ( Sandars, 2009), a strategy to improve judgement, to personally develop, or to contextualize practice ( Chaffey, de Leeuw and Finnigan, 2012), and as a means to identify learning needs, develop the professional identity or build integrated knowledge bases ( Mann, Gordon and MacLeod, 2009).

Lack of coherence in the definition, purposes and goals of reflexivity hinders understanding of medical students and clinicians who have never been introduced to the concept and have a limited practical experience ( Aukes *et al.*, 2007; Aukes *et al.*, 2009; Leung *et al.*, 2010; Chaffey, de Leeuw and Finnigan, 2012; Sandars, Kokotailo and Singh, 2012). This can be an obstacle for medical professors who must try to synthesize information by integrating the multitude of definitions available. In addition, despite the emphasis placed on reflexivity in medical training and continuing medical education, there is a surprising lack of resources to support professors in fostering reflective ability in trainees and learners ( Mann, Gordon and MacLeod, 2009). Some studies have found that clarity in the purpose of the reflective process and reflexive role models are key to facilitate the integration of this skill in trainees and students ( Leung *et al.*, 2010; Chaffey, de Leeuw and Finnigan, 2012). Therefore, synthesizing and simplifying the concept of reflexivity for students and clinical teachers is essential to facilitate the implementation of reflexivity in clinical practice.

### 1.1 Context of the project

Since 2009, reflexive activities in groups have been implemented in the teaching curriculum of the Family Medicine Residency Program at Université Laval. The purposes of these activities are to consolidate the process of reflection on action and to develop reflexive skills in residents of the program. In 2016, the evaluation of the program uncovered some dissatisfactions and questions related to reflexive activities, their relevance and their ability to stimulate a real reflexive process among participants. For instance, the evaluation results revealed a limited understanding of reflexivity and mixed appreciation of the activities in their current format, which were mistakenly designed by participants as pretexts for ventilation episodes, simple/trivial discussions, or masterful teaching. These results highlight vagueness and confusion surrounding the notion of reflexivity and its application in the context of the Family Medicine Residency Program at Université Laval. Following this evaluation, a need to adequately equip clinician teachers and family medicine supervisors to improve their understanding of reflexivity, its practical application in their practice, and ways in which it can be developed among residents was identified.

We conducted this rapid review in the context of a larger project at Université Laval, which aimed to co-develop a relevant tool to support clinical teachers affiliated with the Family Medicine Residency Program in their role of reflexive mentors. The goal of this review is to synthesize recent scientific literature on reflexivity in family medicine and to provide a portray of different conceptions of reflexivity that exist within the field. Results of the review has allowed to frame the contents of the educational tool.

## 2. Methods

We used a rapid review ( Khangura *et al.*, 2012) to quickly synthetize relevant literature on reflexivity in family medicine, which informed the tool development within the context of our larger study. The literature search protocol was developed in consultation with a librarian research specialist and is described using Preferred Reporting Items for Systematic Reviews and Meta-Analyses (PRISMA) ( Moher *et al.*, 2009).

### 2.1 Data sources

We searched six online databases: *PubMed, Embase, PsychInfo, CINHAL, ERIC* and *Education Source.* Based on a systematic approach, we searched each database using a predefined list of keywords related to reflexivity (see Supplementary File 1 for the full search strategy), which was validated by the librarian research specialist. The last search was conducted on the May 25 ^th^, 2017. We imported and collated the results of the database searches using Endnote software version X9.

### 2.2 Studies selection

The entire initial selection of studies based on the titles and abstracts of the corpus was conducted by two reviewers (NTD and LG). Any discrepancies were resolved by consultation with a third reviewer (MCT). To be included in the review, each study had to adhere to the following criteria:

1. be a primary qualitative, quantitative, or mixed methods study published in the last 10 years (May 2007 to May 2017);
2. written in either English or French;
3. conducted in one or many Western countries;
4. describing an intervention aiming to develop reflexivity or studying how family general practitioners or residents implement reflexivity in their current practice.

Studies reporting interprofessional interventions or including numerous specialists were included as long as they also involved family medicine practitioners or students.

### 2.3 Data extraction and analysis

Data was extracted by a first reviewer (NTD) and validated by another (LG), with verification done by the corresponding author (MCT). Data synthesized from the studies included was imported into a spreadsheet with the following column headings: authors, year of publication, study title, definition of reflexivity or associated concepts (where appropriate), usefulness of reflexivity for practice or goals (if mentioned) (See Supplementary File 2). Two team members conducted a thematic analysis (LG, MCT), focusing on the definition of reflexivity and the characteristics of the reflection process involved. The first author (MCT) generated the initial codes and themes related to the different forms of reflexivity, their usefulness/goals and their process. A second coder (LG) validated this analysis. Coding differences were resolved by consensus.

## 3. Results

### 3.1 Rapid review results

**Figure 1.**
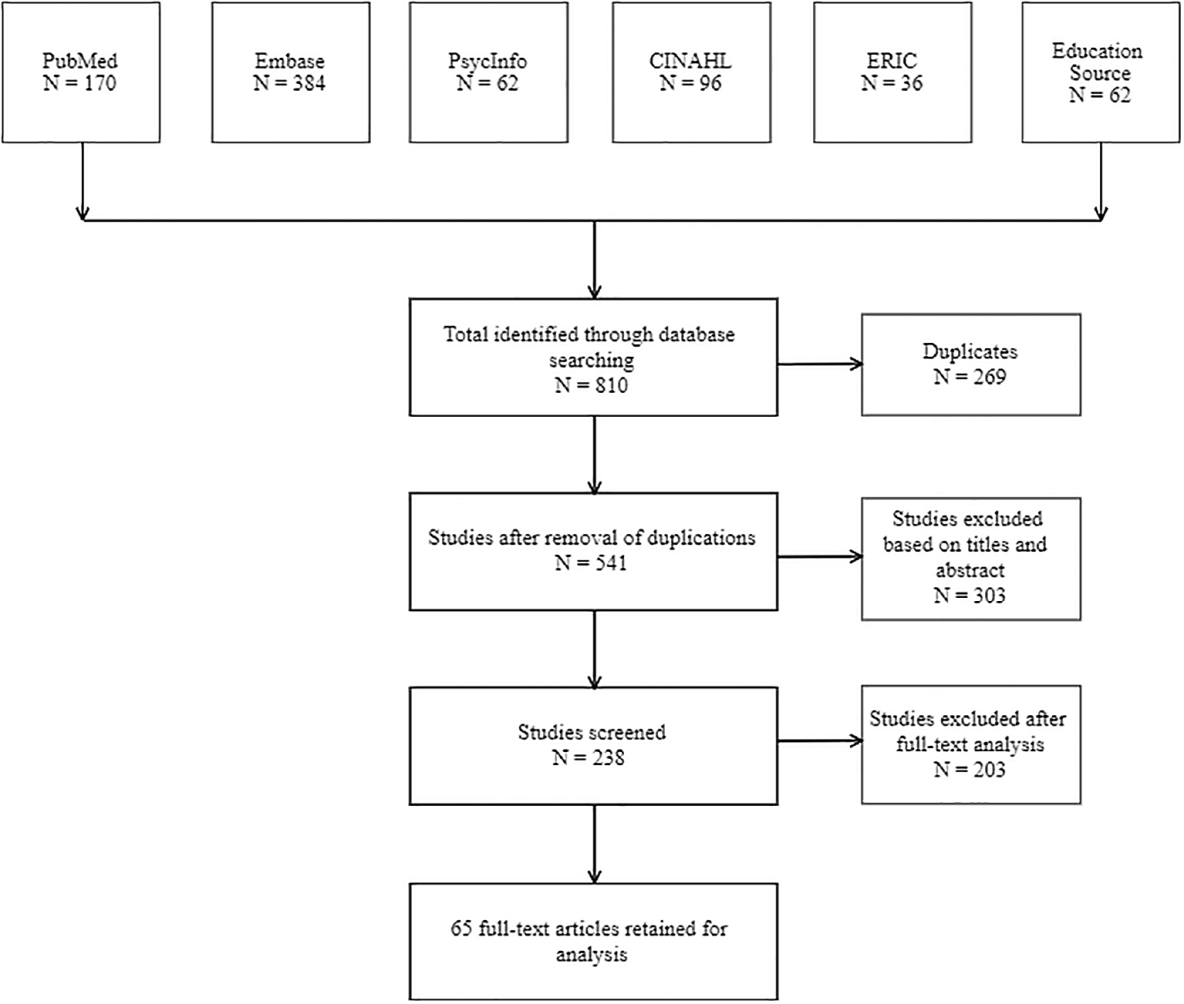
Flow chart for the selection of eligible studies

Our research strategy initially identified 810 studies. After the removal of duplicates (n= 269), the corpus was reduced to 541 studies. The remaining corpus was evaluated based on titles and abstracts and allowed the exclusion of 303 studies. Therefore, the complete texts of 238 studies were accessed, and 65 studies were retained for analysis (see Figure 1).

The different conceptions of reflexivity encountered in the 65 studies (see Supplementary File 3) were analyzed using thematic analysis and regrouped in four main types of reflexivity, with different goals, processes and uses for family medicine practice (see Table 1). The first conception of reflexivity, which we named “clinical reflexivity”, was identified in 33 studies. Clinical reflexivity is identified as a form of reflection initiated by the detection of a clinical error, the identification of a gap in information or professional skills. This type of reflexivity often emerges from a situation where the clinician notices a discrepancy between the desired effects and the actual effects of his actions. The goal of clinical reflexivity is to improve technical and clinical skills by taking the necessary steps to meet educational and professional needs. “Professional reflexivity” is another conception of reflexivity encountered in 40 studies that is closely associated with Schön’s concept of reflection-on-action (Schön, 1984). This kind of reflexivity occurs when a new, ambiguous, or complex situation arises. Being reflexive in these contexts is crucial because it helps to develop professional expertise by creating new patterns of action that will enable the complexity of the practice to be faced. This also creates new schemes of action to draw on that can be integrated with existing repertoires of potential action strategies. A third conception of reflexivity identified in 26 studies is related to introspection and being more aware of feelings, emotions and values in relation to certain situations in professional practice. “Relational reflexivity” is often initiated by the awareness of an emotional state related to a situation experienced. The goal of this type of reflexivity is the development of an empathetic, healthy, and effective therapeutic relationship and process by allowing the healthcare provider to act with compassion and lucidity toward his patients. Finally, “social reflexivity” is a fourth conception of reflexivity reported by 22 studies as a form of reflection on practice sensitive to social considerations, in line with a critical epistemology of practice. This type of reflexivity is initiated by the clinician’s need to make sense of his practice in relation to a broader sociopolitical context by emphasizing the social, ethical, and political aspects of his practice. By so doing, the clinician develops the non-cognitive dimensions of professionalism, such as moral commitment, sense of mission and responsible practice. The four conceptions of reflexivity identified in the analysis helped to develop the formal content of the tool.

**Table 1.** Reflexivi-Tool's content

Type	Trigger of reflexivity	What is it?	Why is it important?	How does it manifest?	Questions to consider
1. Clinical reflexivity	Detection of an error, lack of information, or skill gap	Clinical reflexivity: focuses on improving technical and clinical skills by detecting and correcting errors	It allows professionals to identify their educational needs and take steps to meet them.	1. Professionals notice a discrepancy between the desired effects and the actual effects of their actions. 2. They seek to correct this discrepancy by changing their course of action. 3. Problematic situations are not necessarily considered in relation to previous experiences (non-integrated learning).	What happened? What was the intended outcome and what are the actual results? Why didn't it work? Do I have all the information, technical skills, or clinical skills required to solve this problem? What information or skill do I need to better manage this type of situation in the future? vi) How can I find this information or develop these skills?
2. Professional reflexivity	New, unusual, ambiguous, or complex situation	Professional reflexivity: focuses on developing professional expertise in response to new or complex situations in a professional's practice.	It allows professionals to develop new courses of action based on their experience, which allows them to tackle complex situations in their practice.	1. Professionals identify a problem by changing their initial understanding of it and responding in a way that is different from their usual course of action. 2. This creates a reserve of new models to draw on and that can be constantly renewed in future situations.	What happened? What surprised me in this situation? Why is this situation complex or problematic? Why am I having a hard time working in this situation? What is wrong with my understanding of the problem? How can I rethink this problem? vii) What could I do differently in the future?
3. Relational reflexivity	Strong emotional response to a situation	Relational reflexivity: aims to develop a healthy, effective, and compassionate therapeutic relationship.	It allows professionals to act compassionately and coherently with their patients through a healthier therapeutic relationship.	1. Professionals examine their emotions, values, and beliefs (including their biases) in relation to their patients and the clinical situation. 2. In so doing, they bring to light non-rational elements underlying their judgments and practices. 3. Recognizing the influence of these elements helps professionals dissociate themselves from them and build a healthier, more compassionate therapeutic relationship.	What happened (focus on non-rational elements of the situation)? What emotions does this situation generate? Why do I have a problem with this situation? Which beliefs or values are shaken by this situation? How could my emotions, values, or beliefs have influenced my judgments, attitudes, and actions? What influence does this have on my relationship with this patient? vii) In the future, how can I consider the influence my emotions, values, and beliefs have on my actions?
4. Social reflexivity	The need to make sense of their practice in relation to a wider sociopolitical context	Socially responsible reflexivity aims to develop more responsible, ethical, and moral practices by examining the ethical, social, and political implications of a professional's practice.	It allows professionals to develop non-cognitive aspects of professionalism, such as moral commitment, a sense of purpose, and responsible practices.	1. Professionals question the power dynamics that affect both their actions and the health of their patients. 2. They examine the ethical, social, and political consequences of their actions and practices. 3. In so doing, they develop a new understanding of their experience, their patients' experience, and their professional identity within a broader sociopolitical context.	What happened? What power dynamics are affecting this situation? What are the ethical, social, or political consequences of my actions? Why do we do things this way? How can we do things differently? Which resources can I rely on? vi) What is my social and moral responsibility to my patients and my community or their community?

### 3.2 Development of the tool

The initial version of Reflexivi-Tool (in French *Réflexi-Vite*, literally "Reflect-fast") was developed in collaboration with the Family Medicine Residency Program managers based on local needs identified as well as the conceptual categories drawn from the literature review. The initial version of the tool was refined following a pre-test workshop with typical users (i.e. clinical teachers of the program). Feedback offered by participants of the workshop was used to clarify descriptions in the tool and revise the name of some categories. The tool was designed to support reflexivity in different contexts: clinical, educational or supervisory activities; daily or punctual activities; facilitation as part of an individual or group approach; by users from the same profession (e.g. family medicine) or as part of interprofessional collaboration activities.

The final version of Reflexivi-Tool (see Supplementary File 4) presents four types of reflexivity as previously described (i.e. clinical, professional, relational and social) in a concise and user-friendly way. For each type, the trigger and focus of the reflection process, a role model name (e.g., the empathetic professional referring to relational reflexivity) and a definition is provided (What is it?). There is also a description of the usefulness of each type of reflexivity for practice (Why is it important?) in terms of the skills that it enables. It finally provides a description of the reflection process (How does it manifest?) and a list of question prompts (Questions to consider) that can be used in a supervisory context to support the reflection. Reflexivi-Tool allows for quick understanding of the type of reflexivity generated by a situation. The tool is available in PDF and interactive PDF (optimized for smartphones) formats.

The tool was disseminated to the program’s clinical teachers and residents through a series of four emails from January to May 2019, by an announcement on the online program portal and through a formal presentation to the program’s teaching assistants in September 2018. A series of four short educational videos have also been produced to facilitate the understanding and adoption of the tool. Each video illustrates one of the four types of reflexivity through a staging between a resident and his/her supervisor. These videos have been shared in the emails and on the online program portal.

## 4. Conclusion

Reflexivity is an essential competency of clinicians allowing continued improvement and adaptation of practice in a constantly evolving healthcare system ( Mann, Gordon and MacLeod, 2009; Sandars, 2009; Wald and Reis, 2010; Menard and Ratnapalan, 2013). However, in medical education literature, the concept of reflexivity is inconsistent and ill-defined, with variable purposes and associated processes, which is an important barrier to learning and implementing reflective practices ( Aukes *et al.*, 2007; Aukes *et al.*, 2009; Leung *et al.*, 2010; Chaffey, de Leeuw and Finnigan, 2012). Several authors have highlighted the need to provide clear guidelines regarding the purpose and process of reflexivity, as well as better equipping mentors so they can better facilitate these kinds of skills ( Carr and Carmody, 2006; Chaffey, de Leeuws and Finnigan, 2012). Based on a systematic approach to educational design, this study has allowed the development of a tool that presents and clarifies four main types of reflexivity for medical practice in a concise and user-friendly way. Educational devices and facilitation tools such as Reflexivi-Tool are crucial to support reflective processes that target different dimensions of professionalism, allowing not only improved clinical judgement and continued learning, but also a sense of responsibility and moral commitment in practitioners.

## Take Home Messages

• In the last decade, reflexivity has emerged as a key concept in family medicine. However, this concept of reflexivity is inconsistent and ill-defined in medical education literature, with variable purposes and associated processes.
• We conducted a rapid review to synthesize recent scientific literature on reflexivity in family medicine, which served as a basis to the development of an educational tool to support the learning and teaching of reflexivity.
• The results identify four main types of reflexivity (i.e. clinical, professional, relational, and social reflexivity) with related definitions, goals, processes.

## Notes On Contributors

**Marie-Claude Tremblay** (PhD, MA) is an Assistant Professor in the Department of Family and Emergency Medicine at Université Laval (Québec, Canada). She leads various projects that address cultural safety in healthcare, patient participation in research and training, and reflexivity as a means of transforming health care practices.

**Laurence Garceau** is a doctoral student in clinical psychology at the School of Psychology of Université Laval (Québec, Canada) and a research professional for this team.

**Ndeye Thiab Diouf** (MSc) is a PhD student in community health at Université Laval (Québec, Canada). Her doctoral thesis focuses on reflective practice as a strategy to improve health care professionals’ training on shared decision making in order to foster share decision making implementation and collaborative practice in health care.

**Anne Guichard** (PhD) is an Associate Professor at the Faculty of Nursing of Université Laval (Québec, Canada). Her research interests include practice change in health, program evaluation and knowledge transfer with a focus on population health equity.

**Julien Quinty** (MD, CCMF) is an Assistant Professor at the Department of family medicine and Emergency Medicine at Université Laval (Québec, Canada).

**Chantal Gravel** (MBA, MSc) is a Research and Planning Officer in the Department of Family Medicine and Emergency Medicine at Université Laval (Québec, Canada).

**Christian Rheault** (MD, CCMF) is the Director of the Family Medicine Residency Program and an Assistant Professor at the Department of family medicine and Emergency Medicine at Université Laval (Québec, Canada).

## References

[ref1] Al-Imari L. Yang J. and Pimlott N. (2016) Peer-support writing group in a community family medicine teaching unit: Facilitating professional development. Canadian Family Physician,62(12), pp.e724-e730. 27965348 PMC5154663

[ref2] Aukes L. C. Cohen-Schotanus J. Zwierstra R. P. and Slaets J. P. (2009) The float model: visualizing personal reflection in healthcare. Education for Health (Abingdon, England),22(1), p.210. https://www.educationforhealth.net/text.asp?2009/22/1/210/101561 19953440

[ref3] Aukes L. C. Geertsma J. Cohen-Schotanus J. Zwierstra R. P. (2007) The development of a scale to measure personal reflection in medical practice and education. Medical Teacher,29(2-3), pp.177-182. 10.1080/01421590701299272 17701630

[ref4] Bethune C. and Brown J. B. (2007) Residents’ use of case-based reflection exercises. Canadian Family Physician,53(3), pp.471-476. 17872683 PMC1949082

[ref5] Carr S. and Carmody D. (2006) Experiential learning in women’s health: medical student reflections. Medical Education,40(8), pp.768-774. 10.1111/j.1365-2929.2006.02536.x 16869922

[ref6] Chaffey L. J. de Leeuw E. J. and Finnigan G. A. (2012) Facilitating students’ reflective practice in a medical course: literature review. Education for Health (Abingdon, England),25(3), pp.198-203. 10.4103/1357-6283.109787 23823640

[ref7] D’Cruz H. Gillingham P. and Melendez S. (2007) Reflexivity, its Meanings and Relevance for Social Work: A Critical Review of the Literature. The British Journal of Social Work,37(1), pp.73-90. 10.1093/bjsw/bcl001

[ref8] Davidsen A. S. and Reventlow S. (2011) Different approaches to understanding patients in general practice in Denmark: a qualitative study. British Journal of Guidance & Counselling,39(3), pp.209-226. 10.1080/03069885.2011.552600

[ref9] Frank J. R. (2015) ‘Référentiel de compétences CanMEDS 2015 pour les médecins’ [CanMEDS Competency Framework 2015], Collège royal des médecins et chirurgiens du Canada: Ottawa.

[ref10] George P. Reis S. Dobson M. and Nothnagle M. (2013) Using a learning coach to develop family medicine residents’ goal-setting and reflection skills. Journal of Graduate Medical Education,5(2), pp.289-293. 10.4300/JGME-D-12-00276.1 24404275 PMC3693696

[ref11] Gustafsson C. and Fagerberg I. (2004) Reflection, the way to professional development?. Journal of Clinical Nursing,13(3), pp.271-280. 10.1046/j.1365-2702.2003.00880.x 15009329

[ref12] Issitt M. (2003) Reflecting on reflective practice for professional education and development in health promotion. Health Education Journal,62(2), pp.173-188. 10.1177/001789690306200210

[ref13] Khangura S. Konnyu K. Cushman R. Grimshaw J. (2012) Evidence summaries: the evolution of a rapid review approach. Systematic Reviews,1(1), p.10. 10.1186/2046-4053-1-10 22587960 PMC3351736

[ref14] Laidlaw T. S. Kaufman D. M. Sargeant J. MacLeod H. (2007) What makes a physician an exemplary communicator with patients?. Patient Education and Counseling,68(2), pp.153-160. 10.1016/j.pec.2007.05.017 17628387

[ref15] Leung K. H. Pluye P. Grad R. and Weston C. (2010) A reflective learning framework to evaluate CME effects on practice reflection. Journal of Continuing Education in the Health Professions,30(2), pp.78-88. 10.1002/chp.20063 20564716

[ref16] Mann K. Gordon J. and MacLeod A. (2009) Reflection and reflective practice in health professions education: a systematic review. Advances in Health Sciences Education: Theory and Practice,14(4), pp.595-621. 10.1007/s10459-007-9090-2 18034364

[ref17] Menard L. and Ratnapalan S. (2013) Reflection in medicine: Models and application. Canadian Family Physician,59, p.105. 23341668 PMC3555667

[ref18] Mezirow J. (1990) Fostering critical reflection in adulthood: A guide to transformative and emancipatory learning. 1sted. San Francisco: Jossey-Bass.

[ref19] Moher D. Liberati A. Tetzlaff J. Altman D. G. (2009) Preferred reporting items for systematic reviews and meta-analyses: the PRISMA statement. Annals of Internal Medicine,151(4), pp.264-269. 10.7326/0003-4819-151-4-200908180-00135 19622511

[ref20] Page M. J. McKenzie J. E. Bossuyt P. M. Boutron I. (2021) Updating guidance for reporting systematic reviews: development of the PRISMA 2020 statement. Journal of Clinical Epidemiology,134, pp.103-112. 10.1016/j.jclinepi.2021.02.003 33577987

[ref21] Sandars J. (2009) The use of reflection in medical education: AMEE Guide No. 44. Medical Teacher,31(8), pp.685-695. 10.1080/01421590903050374 19811204

[ref22] Sandars J. Kokotailo P. and Singh G. (2012) The importance of social and collaborative learning for online continuing medical education (OCME): directions for future development and research. Medical Teacher,34(8), pp.649-652. 10.3109/0142159X.2012.687847 22830322

[ref23] Tannenbaum D. (2009) ‘Rôles CanMEDS - Médecine familiale’ [CanMEDS - Family Medicine], Collège des médecins de famille du Canada, Section des enseignants en médecine familiale, Groupe de travail sur la révision du cursus: Mississauga, ON.p.26.

[ref24] Wald H. S. and Reis S. P. (2010) Beyond the Margins: Reflective Writing and Development of Reflective Capacity in Medical Education. Journal of General Internal Medicine,25(7), pp.746-749. 10.1007/s11606-010-1347-4 20407840 PMC2881974

