# Supplementary material for: Improving Understanding of Reflexivity in Family Medicine: Development of an Educational Tool Based on a Rapid Review
Source: MedEdPublish (2016). 2021 Jul 19;10:181. [Version 1] doi: 10.15694/mep.2021.000181.1 (PMC10939579; doi:10.15694/mep.2021.000181.1)
Supplement: Peer review history

# Reviewer report: Reviewer response for version 1

**DOI:** 10.21956/mep.20440.r31660

**Published:** 2021-10-12

**Authors:** Balakrishnan Nair

**Affiliations:** 1 Centre for Medical Professional Development and University of Newcastle

This review has been migrated. The reviewer awarded 4 stars out of 5 I enjoyed reading this article; it has expanded my knowledge of reflection in medical education. The authors have done a comprehensive, rapid review on the topic and come up with new concepts of reflection in medical practice. The original concept was on reflection in practice and reflection on practice described by Schon. The authors have come up with innovative concepts of reflection in professional, social and relational co. The take-home message for me is the Reflexivi Tool

Reviewer Expertise:

NA

No decision status is available

# Reviewer report: Reviewer response for version 1

**DOI:** 10.21956/mep.20440.r31661

**Published:** 2021-09-03

**Authors:** BALAJI ARUMUGAM

**Affiliations:** 1 TAGORE MEDICAL COLLEGE AND HOSPITAL

This review has been migrated. The reviewer awarded 4 stars out of 5 A well written review of literature on reflexivity. Kudos to the author for their wonderful work on this very important and innovative topic. Thematic analysis of 65 studies finalized by literature search of quantitative, qualitative and mixed method studies were included. An educational tool on Reflexitivity was develioped and designed based on the thematic analyis and the types of reflexivity in the final version of Reflexivi-Tool presents four types of reflexivity as described i.e. clinical, professional, relational and social in a concise and user-friendly way. Good work and keep it up. The contents and the subheadings on the various types of reflexivity are important aspects which can be utilized by the physicians to improve on reflexivity.

Reviewer Expertise:

NA

No decision status is available

# Reviewer report: Reviewer response for version 1

**DOI:** 10.21956/mep.20440.r31662

**Published:** 2021-07-19

**Authors:** Daniel Xu

**Affiliations:** 1 Curtin Medical School

This review has been migrated. The reviewer awarded 3 stars out of 5 A good review of the concept of "reflection" in terms of family medicine training perspective, it'll be better if it can discuss in terms of the new combined domains of GP training: Bio-Psycho-Social-Political-Economical-Spiritual. Expand on the practical aspect of the the tool.

Reviewer Expertise:

NA

No decision status is available
